# CtBP determines ovarian cancer cell fate through repression of death receptors

**DOI:** 10.1038/s41419-020-2455-7

**Published:** 2020-04-24

**Authors:** Boxiao Ding, Fang Yuan, Priyadarshan K. Damle, Larisa Litovchick, Ronny Drapkin, Steven R. Grossman

**Affiliations:** 1 Department of Internal Medicine, Virginia Commonwealth University, Richmond, VA 23298 USA; 2 VCU Massey Cancer Center, Virginia Commonwealth University, Richmond, VA 23298 USA; 3 Ovarian Cancer Research Center, Department of Obstetrics and Gynecology, University of Pennsylvania, Philadelphia, PA 19104 USA

**Keywords:** Ovarian cancer, Oncogenesis

## Abstract

C-terminal binding protein 2 (CtBP2) is elevated in epithelial ovarian cancer, especially in the aggressive and highly lethal subtype, high-grade serous ovarian cancer (HGSOC). However, whether HGSOC tumor progression is dependent on CtBP2 or its paralog CtBP1, is not well understood. Here we report that CtBP1/2 repress HGSOC cell apoptosis through silencing of death receptors (DRs) 4/5. CtBP1 or 2 knockdown upregulated DR4/5 expression, and triggered autonomous apoptosis via caspase 8 activation, but dependent on cell-type context. Activation of DR4/5 by CtBP1/2 loss also sensitized HGSOC cell susceptibility to the proapoptotic DR4/5 ligand TRAIL. Consistent with its function as transcription corepressor, CtBP1/2 bound to the promoter regions of DR4/5 and repressed DR4/5 expression, presumably through recruitment to a repressive transcription regulatory complex. We also found that CtBP1 and 2 were both required for repression of DR4/5. Collectively, this study identifies CtBP1 and 2 as potent repressors of DR4/5 expression and activity, and supports the targeting of CtBP as a promising therapeutic strategy for HGSOC.

## Introduction

High-grade serous ovarian cancer (HGSOC), which accounts for up to 70% of epithelial ovarian cancer, is a complex, heterogeneous disorder, and remains the most lethal gynecologic malignancy^1–3^. The mainstay of treatment for HGSOC is the combination of surgical cytoreduction and chemotherapy. Although initially sensitive to chemotherapy, the majority of HGSOC gradually acquire genomic change, and eventually relapse as incurable disease^4–6^. Therefore, understanding the genetic dependency and molecular features of HGSOC proliferation could improve the development of effective therapeutic strategies^7^. Recent studies have shown that C-terminal binding protein-1 and -2 (CtBP1/2) are overexpressed in ovarian cancer, and CtBP2 expression is correlated with poor prognosis^8,9^. Analysis of The Cancer Genome Atlas database also revealed a high frequency of CtBP1/2 gene amplification in HGSOC^10^.

Human CtBP1 and 2 are highly conserved, sharing 78% amino acid identity and 83% similarity^11,12^. Following the earliest discovery that CtBP1 binds to the C-terminus of the human adenovirus E1A protein^13,14^, numerous studies have shown that CtBP proteins function as transcriptional coregulators participating in embryonic development as well as adult biological processes^15^. The detailed mechanism underlying the function of CtBP proteins in transcriptional regulation is not fully understood, but presumably occurs through the interaction with a complex of transcription factors and chromatin modifiers at CtBP target genes^9,10^. Indeed, CtBP proteins bind to a variety of transcriptional factors containing a consensus PxDLS peptide motif and are targeted to promoters via interaction with sequence specific DNA binding proteins, such as ZEB1, KLF8, Evi-1, and others^16–18^.

Accumulating evidence has supported the notion that CtBP is pro-tumorigenic, as inhibition of CtBP results in apoptosis through induction of Noxa, PUMA, and Bik in a variety of cancer cell types^19,20^, indicating CtBP overexpression could be a mechanism underlying the bypass of apoptosis, a key hallmark of cancer. In addition to CtBP itself, many CtBP interacting factors are also involved in oncogenic processes, such as epithelial–mesenchymal transition^21^, cancer metastasis, and apoptosis^20,22^. Relative to a potential role for CtBP as a key oncogenic driver or dependency, our previous studies have shown that CtBP2 is a dependency for APC mutated neoplasia in the *Min* mouse intestinal polyposis model of human Familial Adenomatous Polyposis^23^. We further demonstrated that CtBP2 haploinsufficiency reduced tumor initiating cell (TIC) abundance in APC^min/+^ intestines, suggesting the oncogenic role of CtBP2 in intestinal neoplasia relates to its promotion of TIC activities^24^. These findings were more recently mirrored by similar findings in a mouse model of human pancreatic adenocarcinoma (PDAC), where CtBP2 deficiency slowed tumor growth, abrogated metastases, and severely attenuated expression of TIC markers^25^.

Here, we investigated the potential CtBP dependency of HGSOC. We demonstrated that CtBP1/2 RNAi depletion induced activation of caspase 8 via death receptor DR4 and/or DR5 induction, resulting in cell-autonomous apoptosis or enhanced sensitivity to TRAIL, depending on cell type. CtBP1 and 2 bound to the promoters of the DR4/5 genes and coordinately suppressed their expression. Our findings uncover an antiapoptotic mechanism of CtBP in HGSOC with potential implications for future novel therapies.

## Materials and methods

### Cell culture and reagents

Human ovarian cancer cell lines were cultured in either RPMI 1640 (for KURAMOCHI, OVSAHO, SKOV3, HEY, and A2780), or DMEM (for OVCA429 and CAOV3) supplemented with 10% fetal bovine serum, 0.1 mg/mL penicillin, and 0.1 mg/mL streptomycin. SKOV3 and CAOV3 cells were obtained from ATCC; KURAMOCHI and OVSAHO cells were a gift from Dr. Gottfried Konecny (UCLA, Los Angeles, CA); HEY, A2780, and OVCA429 cells were a gift from X. Fang (VCU, Richmond, VA). Z-DEVD-FMK was purchased from Sigma. Recombinant human TRAIL was purchased from Gemini Bio-products.

### RNAi

All shRNA constructs were obtained from Sigma: pLKO.1-shCtrl (#1 SHC016, and #2 SHC002), pLKO.1-shCtBP1 (SHCLND-NM_001328, #1 TRCN0000285086, and #2 TRCN0000273842), and pLKO.1-shCtBP2 (SHCLND-NM_001329, #1 TRCN0000013744 and #2 TRCN0000013745). Lentivirus-mediated shRNA were produced by cotransfection of HEK293T cells with pLKO.1 constructs along with the pCMV delta R8.2 packaging plasmid and pCMV-VSV-G. pCMV delta R8.2 was a gift from D. Trono (Addgene plasmid #12263), pCMV-VSV-G was a gift from B. Weinberg (Massachusetts Institute of Technology, Cambridge, MA) (Addgene plasmid # 8454; http://n2t.net/addgene:8454; RRID:Addgene_8454). siRNA oligos were purchased from Thermo Fisher Scientific: siCtrl (#4390843), siCaspase 8 (#s2427), siTNFRSF10A (DR4) (#s16764), and siTNFRSF10B (DR5) (#s16756). siRNA reverse transfection was performed using Lipofectamine RNAiMAX (Invitrogen) as per manual.

### Western blot and immunoprecipitation

Cells were washed with cold PBS and lysed in RIPA buffer (25 mM Tris-HCl, pH = 7.5, 150 mM NaCl, 0.1% Nonidet P-40, 0.5% sodium deoxycholate, and 0.1% SDS) supplemented with protease inhibitor cocktail (Sigma) and Phosphatase Inhibitor Cocktail 2 and 3 (Sigma). The lysates were cleared by centrifugation at 13,800 × *g* for 15 min, and then subjected to SDS-PAGE and immunoblotting. For immunoprecipitation, cells were lysed in TNTE buffer (50 mM Tris-HCl, pH = 7.5, 150 mM NaCl, 1% Triton X-100, 1 mM EDTA, and protease inhibitors). The whole cell lysates were incubated with Protein A/G PLUS-Agarose (sc-2003, Santa Cruz Biotechnology) and relevant antibodies overnight at 4 °C. Following incubation, agarose beads were washed 3 times in TNTE buffer and heated to 95 °C for 5 min to elute proteins. Protein elution was analyzed by standard western blot. The following antibodies were used: anti-CtBP1 (#612042, BD Biosciences), anti-CtBP2 (#612044, BD Biosciences), anti-caspase 8 (#9746, Cell Signaling Technology, [CST]), anti-caspase 9 (#9502, CST), anti-caspase 3 (#9662, CST), anti-caspase 7 (#9492, CST), anti-PARP1 (sc-53643, Santa Cruz Biotechnology), anti-TNFR1(#3736, CST), anti-FAS (#4233, CST), anti-DR4 (#42533, CST), anti-DR5 (#8074, CST), and anti-GAPDH (sc-32233, Santa Cruz Biotechnology). The following antibodies were validated for immunoprecipitation and ChIP assay: normal rabbit IgG (#2729, CST), anti-CtBP1 (#8684, CST), and anti-CtBP2 (#13256, CST).

### Real-time PCR

Total RNA was extracted using Trizol reagent (Invitrogen). cDNA was synthesized using RevertAid reverse transcriptase (#EP0441, Thermo Scientific) and Oligo(dT)_18_ (#SO131, Thermo Scientific) and qPCR was performed using the iTaq universal SYBR green supermix (Bio-Rad). Actin beta (ACTB) was used for normalization in all qPCR assays. Fold changes were analyzed by the 2^−ΔΔCT^ method for relative quantification. The primers used for qPCR were: TNFRSF10A: 5′-ACCTTCAAGTTTGTCGTCGTC-3′ and 5′-CCAAAGGGCTATGTTCCCATT-3′; TNFRSF10B: 5′-GCCCCACAACAAAAGAGGTC-3′ and 5′-AGGTCATTCCAGTGAGTGCTA-3′; FAS: 5′-TCTGGTTCTTACGTCTGTTGC-3′ and 5′-CTGTGCAGTCCCTAGCTTTCC-3′; TNFR1: 5′-TCACCGCTTCAGAAAACCACC-3′ and 5′-GGTCCACTGTGCAAGAAGAGA-3′, and ACTB: 5′-CACCATTGGCAATGAGCGGTTC-3′ and 5′-AGGTCTTTGCGGATGTCCACGT-3′.

### Cell viability, apoptosis assay, and caspase activity assay

Cell viability was assessed using Trypan blue exclusion assay and the Cell Counting Kit-8 (CCK-8) (#96992, Sigma). Apoptosis assay was performed using FITC-annexin V and propidium Iodide (PI) double staining as per FITC-Annexin V Apoptosis Detection Kit I (#556547, BD Biosciences). Caspase-8, or caspase-3/7 activity were monitored using the Caspase-Glo 8 or Caspase-3/7 assay kits (Promega). Measurement was done using the Glomax multi detection system (Promega).

### Enzymatic ChIP and reChIP assays

Enzymatic chromatin immunoprecipitation (ChIP) was performed as per SimpleChIP enzymatic chromatin IP kit (#9003, CST) instruction with minor revisions^26^. Briefly, four million cells were sequentially crosslinked with 2 mM disuccinimidyl glutarate (DSG, #20593, Thermo Scientific) for 45 min at room temperature for protein–protein fixation followed by treatment with 1% formaldehyde (w/v) (15 min at room temperature) for protein-DNA fixation^27^. The crosslinked cells were quenched by 0.125M glycine and then lysed^26^. The nuclear lysates were incubated with micrococcal nuclease to digest chromatin into 150–900 bp DNA/protein fragments. The soluble DNA/protein fragments were immunoprecipitated with magnetic beads prebound with either normal rabbit IgG (#2729, CST), anti-CtBP1 (#8684, CST) or anti-CtBP2 (#13256, CST). The antibodies used for ChIP assay were validated as CtBP1 or CtBP2-specific by Western blot. After extensive washing, chromatin was eluted from antibody/protein G beads and incubated with proteinase K at 65 °C for 2 h to reverse cross-links. DNA purification was performed using phenol:chloroform:isoamyl alcohol (25:24:1, v/v). qPCR was performed using the iTaq universal SYBR green supermix (Bio-Rad) to quantify DNA. The sequences of all primers are listed in Supplementary Table S1. ChIP enrichment of the targeted promoter amplicon was calculated with the followed equation: percent input = 2% × 2^(C[T] 2%input sample − C[T] IP sample)^, where C[T] is Ct value. For reChIP, the first ChIP was performed with anti-CtBP2 antibody crosslinked with protein G beads and 40 million cells. The chromatin/protein eluted from the first ChIP was immunoprecipitated with anti-CtBP1 antibody and analyzed similarly to the first ChIP.

### Statistical analysis

Data were presented as mean values ± 1 standard deviation (SD). Statistical significance was examined by two-tailed Student *t* test using GraphPad Prism version 8.0.0 for Windows (GraphPad Software, San Diego, California USA, www.graphpad.com).

## Results

### CtBP loss triggers caspase 8-dependent apoptosis

**Fig. 1:**
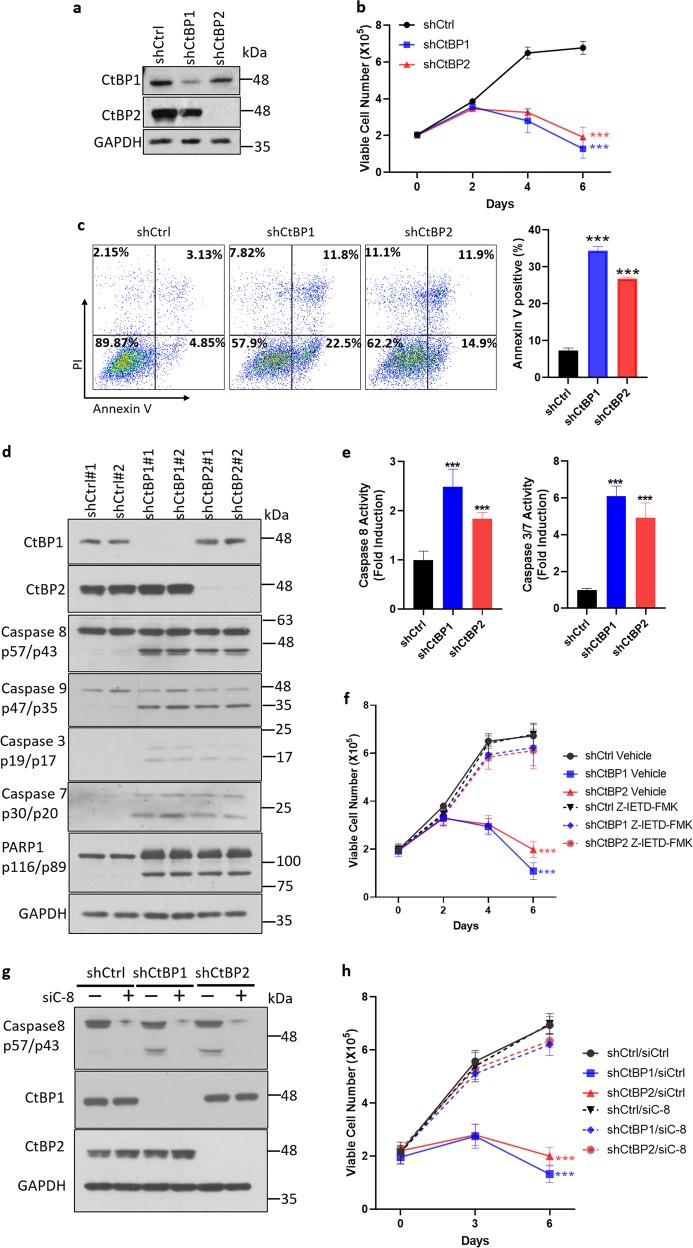
CtBP loss triggers caspase 8-dependent apoptosis. **a**–**e** KURAMOCHI cells were infected with indicated shRNAs. CtBP RNAi efficiency was analyzed by western blotting (**a**). Viable cells were counted by Trypan blue exclusion assay. ***p < 0.001 as compared with shCtrl group (**b**). Four days post infection, cell apoptosis was analyzed by Annexin V/PI staining (left panel); percentage of Annexin V positive apoptotic cells is shown in the bar graph (right panel), ***p < 0.001 as compared with shCtrl group (**c**). Cleaved caspases and PARP1 were analyzed by western blotting (**d**) and caspase 8 and caspase 3/7 activities were determined by enzymatic assay (**e**), ***p < 0.001 as compared with shCtrl group. **f** KURAMOCHI cells, infected with lentiviral shRNA’s as indicated, were treated with vehicle or Z-IETD-FMK (20 µM) for 6 days. Viable cells were counted by Trypan blue assay at indicated time points, ***p < 0.001 as compared with shCtrl group. **g**, **h** KURAMOCHI cells were transfected with siRNAs followed by infection with shRNAs. Cleaved caspase 8 was examined by western blotting (**g**), and viable cells were counted at indicated time points by Trypan blue assay (**h**). ***p < 0.001 as compared with shCtrl group.

Based on the prior demonstration of CtBP1/2 overexpression in ovarian cancer, and poor prognosis related to CtBP2 expression^8,9^, we investigated the cellular CtBP dependency of HGSOC. We knocked down CtBP1 or 2 using lentivirus-based shRNAs in KURAMOCHI cells (Fig. 1a), which are highly genetically representative of HGSOC^28–32^, followed by analysis of cell viability over 6 days in culture. Indeed, we observed a dramatic inhibition of cell growth and loss of viability when cells were treated with lentiviral shCtBP1 or shCtBP2, as compared with the effect of control shRNA (Fig. 1b). We next investigated whether loss of viability related to CtBP1/2 knockdown was due to apoptosis by subjecting KURAMOCHI cells exposed to control, CtBP1 or 2 shRNA to analysis with Annexin V and propidium iodide (PI), measuring apoptosis and cell viability, respectively (Fig. 1c). Indeed, KURAMOCHI cells exhibited robust induction of apoptosis after CtBP1 or 2 knockdown (fourfold and threefold induction, respectively; *p* < 0.001; Fig. 1d).

Sequential activation of caspase cascades, and ultimately PARP cleavage, are the key events in both the intrinsic and extrinsic apoptosis pathways^33^. To gain insight into the mechanism underlying apoptosis after CtBP depletion, we screened proteolytic caspases −3, −7, −9, −8 as well as PARP1 for activation and cleavage after CtBP1 or 2 knockdown in KURAMOCHI cells using both an immunoblot assay for caspase cleavage (Fig. 1d) and caspase enzymatic assays for either caspase 8 or caspases 3 and 7 (Fig. 1e). Two shRNAs each targeting CtBP1 or CtBP2 were used to exclude off-targeting effects. Both shRNAs for CtBP1 and 2 showed similar knockdown efficiency and similar effects on robustly inducing caspase 3/7/8 and PARP1 cleavages, indicating that CtBP1 or 2 knockdown specifically induced activation of caspase cascades (Fig. 1d, e). Of note, caspase 8, which is upstream of other caspases and stands at the initiating step of the extrinsic apoptosis pathway^34^, was clearly cleaved (Fig. 1d, e), indicating activation of caspase 8 is likely the critical event initiating CtBP loss-induced apoptotic cell death. Addition of Z-IETD-FMK, a specific caspase 8 inhibitor, after CtBP1/2 knockdown, completely abrogated cell death in shCtBP1 or 2-expressing cells (Fig. 1f), and indeed restored normal cell growth, consistent with caspase 8 serving as the initiating caspase in the apoptotic caspase activation cascade after CtBP loss. We also knocked down caspase 8 by siRNA prior to CtBP shRNA infection (Fig. 1g), and analyzed cell viability. As expected, caspase 8 depletion rescued CtBP shRNA-induced cell death (Fig. 1h). Taken together, we conclude that CtBP deficiency induces apoptosis in KURAMOCHI HGSOC cells via caspase 8 activation.

### CtBP loss activates caspase 8 via death receptor 4

Canonical extrinsic apoptosis is initiated by ligands binding to their respective death receptors (DRs) such as FAS, TNFR1, death receptor 4 (DR4), and DR5^35^. Apoptotic signals are then transduced via receptor oligomerization and the adaptor protein FADD, which recruits caspase 8, leading to caspase 8 activation by autocatalysis^36^. More recent evidence points to cell-autonomous activation of DRs and signaling to caspase 8 in a ligand independent manner^37–39^. Having shown that CtBP controls apoptosis through caspase 8, we next asked how caspase 8 is activated after CtBP loss.

**Fig. 2:**
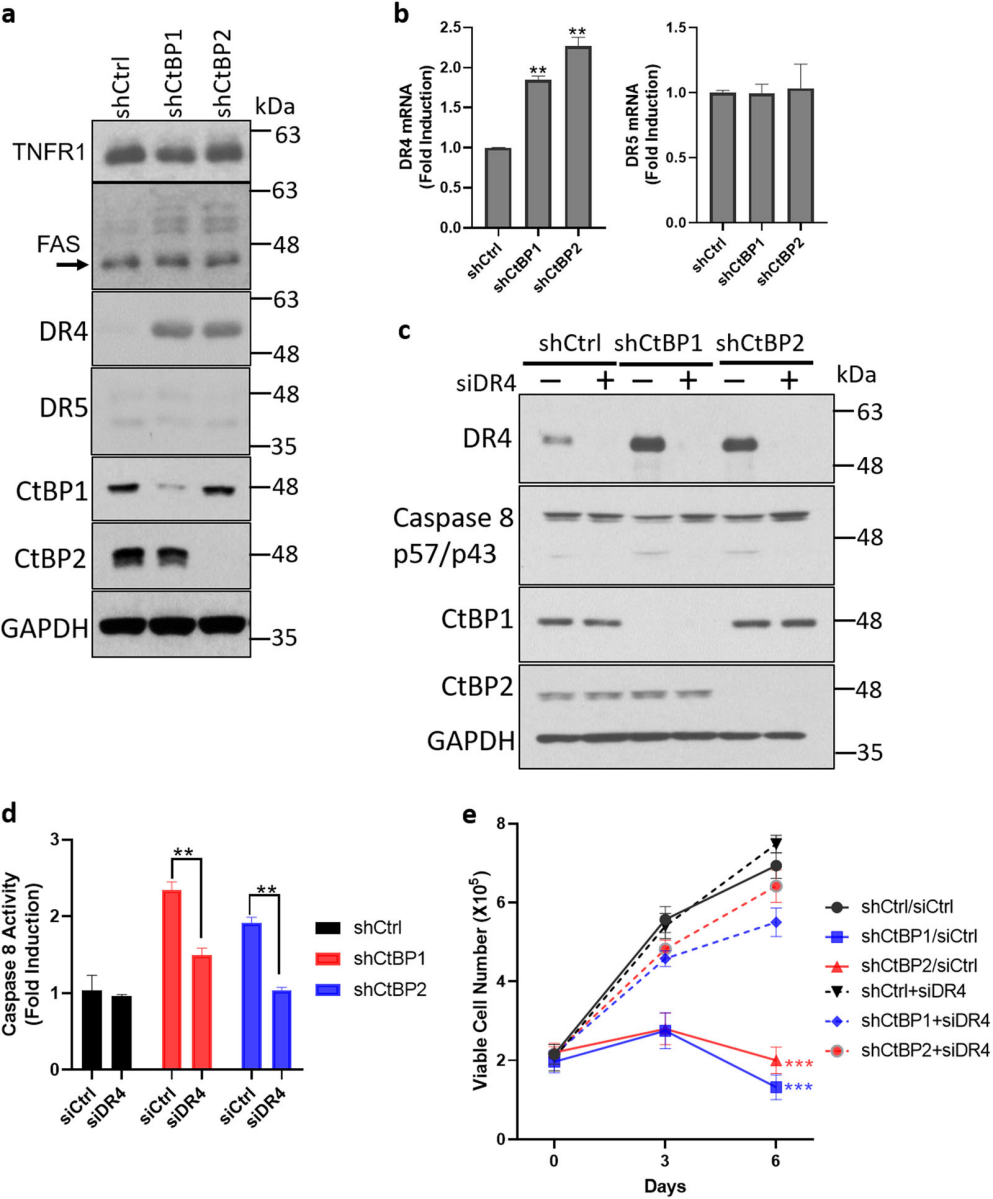
CtBP loss activates caspase 8 via death receptor 4. **a**, **b** KURAMOCHI cells were infected with indicated shRNAs. Cell death receptor expression was examined by western blotting (**a**), DR4 and DR5 mRNA levels were examined by qPCR (**b**), **p < 0.01 as compared with shCtrl group. Arrow in FAS blot points to unmodified FAS, and higher molecular weight forms are consistent with known glycosylation^58^. **c**–**e** KURAMOCHI cells were treated with indicated siRNAs along with shRNAs. Cleaved caspase 8, and the knockdown efficiency of DR4 and CtBP1/2 was determined by immunoblotting (**c**); caspase 8 activity was examined by enzymatic assay (**d**), **p < 0.01 as compared with shCtrl group; and viable cells were counted by Trypan blue assay at indicated time points (**e**). ***p < 0.001 as compared with shCtrl group.

For this purpose, we examined the expression of DRs in KURAMOCHI cells expressing shRNAs for CtBP1 or 2. Interestingly, we found that DR4 was elevated at both protein and mRNA levels after CtBP1 or 2 knockdown, whereas TNFR1, FAS, and DR5 remained unaltered (Fig. 2a, b). We also observed that endogenous TRAIL expression was not affected after CtBP1 or 2 knockdown (Supplementary Fig. S1a). Knocking down endogenous TRAIL did not block or diminish CtBP shRNA-induced cell death (Supplementary Fig. S1b, c). Furthermore, the addition of exogenous TRAIL did not sensitize or enhance cell death after CtBP1/2 loss, despite DR4 upregulation (Supplementary Fig. S2). These data collectively indicated a possible direct TRAIL-independent link between DR4 elevation and caspase 8 activation. To determine if caspase 8 activation after CtBP1 or 2 depletion was dependent on DR4 induction, we simultaneously depleted DR4 and CtBP1/2 by RNAi (Fig. 2c) and found that caspase 8 activation was abrogated after DR4 knockdown, as evidenced by the absence of caspase 8 cleavage (Fig. 2c, d). In parallel, CtBP1/2 loss-induced cell death was abrogated by DR4 depletion (Fig. 2e). These data collectively suggest that CtBP1 or 2 loss led to not only induction of DR4 expression, but also cell-autonomous activation of DR4, which activated caspase 8 and consequently the downstream caspase cascade, resulting in cell apoptosis.

### CtBP represses DR4 and DR5 depending on cell context

**Fig. 3:**
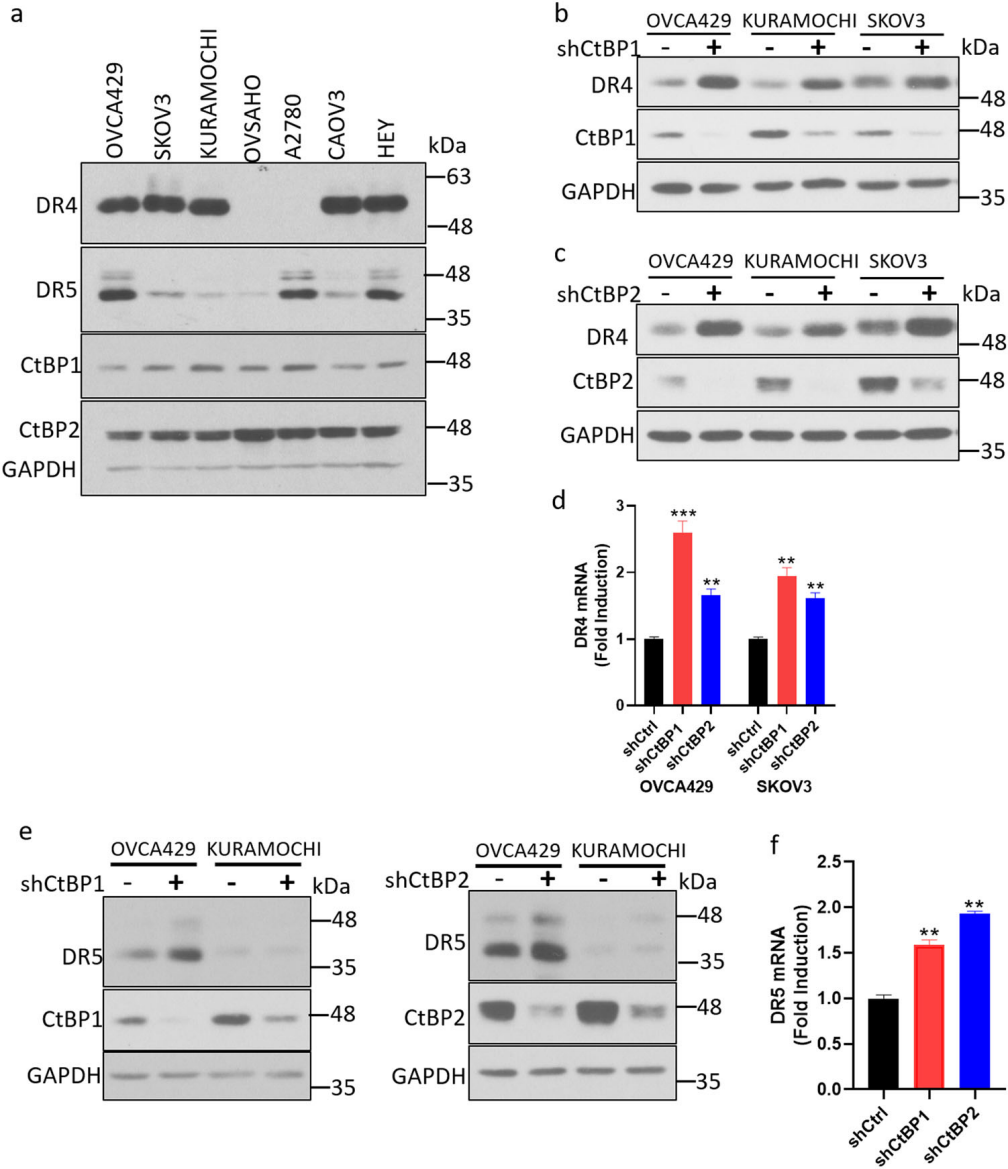
CtBP represses DR4 and DR5 depending on cell context. **a** DR4 and DR5 protein levels were examined by western blotting in indicated HGSOC cell lines. **b**–**d** OVCA429, KURAMOCHI, and SKOV3 were infected with indicated shRNAs. DR4 protein was examined by western blotting (**b**, **c**), mRNA level of DR4 was analyzed by qPCR (**d**), **p < 0.01; ***p < 0.001 as compared with shCtrl group. **e**, **f** OVCA429 and KURAMOCHI cells were infected with indicated shRNAs, and DR5 protein level was examined by western blotting (**e**), DR5 mRNA level in OVCA429 cells was analyzed by qPCR (**f**), **p < 0.001 as compared with shCtrl group.

Previous studies have revealed multilevel controls of DR4/5 expression in different types of HGSOC cells^40,41^. Although we did not observe a change in DR5 expression in KURAMOCHI cells after CtBP1 or 2 knockdown, it is possible that the particular cell context of KURAMOCHI might mask the effect of CtBP on DR5 expression. To better understand how cell context affects CtBP regulation of DRs, we assessed the steady-state levels of DR4/5 and CtBP1/2 in a panel of additional molecularly validated HGSOC cell lines (OVSAHO, CAOV3, HEY, and OVCA429), as well as two additional reference ovarian cancer cell lines (SKOV3 and A2780) which have been used extensively in the literature but molecular studies are unable to classify^31,42^. As shown in Fig. 3a, KURAMOCHI cells as well as three other cell lines (SKOV3, OVSAHO, and CAOV3) exhibited very low DR5 expression, while OVSAHO and A2780 cell lines exhibited no detectable expression of DR4. All of the tested cell lines expressed similar levels of CtBP1 and CtBP2. Expression of DR4 was further enhanced in OVCA429 and SKOV3 cells when CtBP1 or 2 was knocked down (Fig. 3b–d). Remarkably, knockdown of CtBP1/2 caused DR5 upregulation in OVCA429 cells as well (Fig. 3e, f). However, we did not observe any alteration of DR4/5 in CAOV3 and Hey cell lines (Supplementary Fig. S3). Taken together, these results indicate that among HGSOC cell lines, CtBP represses DR4 and DR5 differentially, and in a cell-type dependent manner.

### CtBP depletion sensitizes cells to TRAIL

**Fig. 4:**
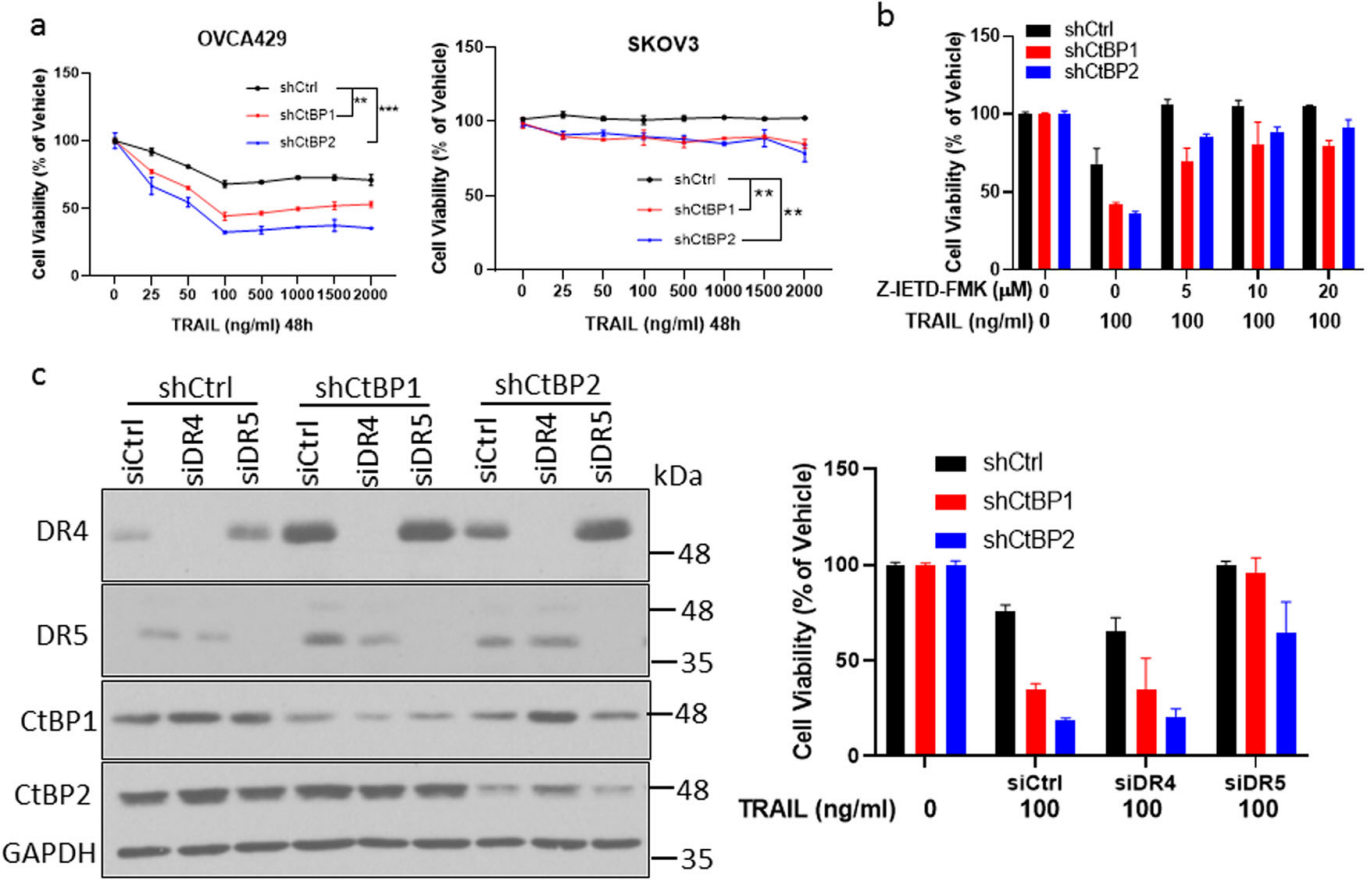
CtBP depletion sensitizes cells to TRAIL treatment. **a** OVCA429 and SKOV3, after infection with indicated shRNAs, were treated with vehicle or TRAIL for 48 h, and relative viable cell number determined by colorimetric assay, **p < 0.01; ***p < 0.001 as compared with shCtrl group. **b** OVCA429 cells, infected with indicated shRNAs, were treated with vehicle or a combination of Z-IETD-FMK and TRAIL for 48 h, and relative viable cell number determined as in (**a**). **c** OVCA429 cells were transfected with siRNAs overnight, then infected with the indicated shRNAs. Left panel: western blotting showing the knockdown efficiency of indicated protein; Right panel: cells were treated with TRAIl for 48 h, and relative viable cell number was assessed as in (**a**).

The induction of DR4 and/or DR5 after CtBP1/2 knockdown in OVCA429 and SKOV3 cells was not accompanied by loss of cell viability as was observed in KURAMOCHI cells (Fig. 1b). Given that DR4/5 signal apoptosis in response to engagement by TRAIL ligand, we next tested whether the fate of these cells was altered when exposed to TRAIL with or without CtBP1 or 2 depletion. Consistent with previous reports^43,44^, OVCA429 cells were modestly susceptible to TRAIL-induced loss of viability (25% reduction at 100 ng/ml compared with vehicle), but depletion of CtBP1/2 substantially sensitized TRAIL-induced loss of viability (50–60% reduction at 100 ng/ml compared with vehicle; Fig. 4a). In contrast, SKOV3 cells were completely resistant to TRAIL, and CtBP1/2 depletion only slightly sensitized SKOV3 to TRAIL-induced loss of viability (15% decrease at 100 ng/ml; Fig. 4a). As expected, caspase 8 inhibitor, Z-IETD-FMK blocked loss of viability induced by TRAIL in OVCA429 cells (Fig. 4b), consistent with a canonical caspase 8-dependent extrinsic apoptotic mechanism induced by TRAIL.

We next knocked down DR4 or 5 along with CtBP1 or 2 in OVCA429 cells to determine if TRAIL-induced sensitization to CtBP1/2 depletion was DR4 or 5 dependent (Fig. 4c). Notably, we observed that TRAIL-induced cell death was markedly diminished by siDR5, but not siDR4 (Fig. 4c), indicating the predominant role of DR5 in transducing TRAIL signal in these cells. Collectively, these results supported the notion that CtBP controls the susceptibility to TRAIL in OVCA429 cells via DR5.

### CtBP binds to DR4/5 promoters

**Fig. 5:**
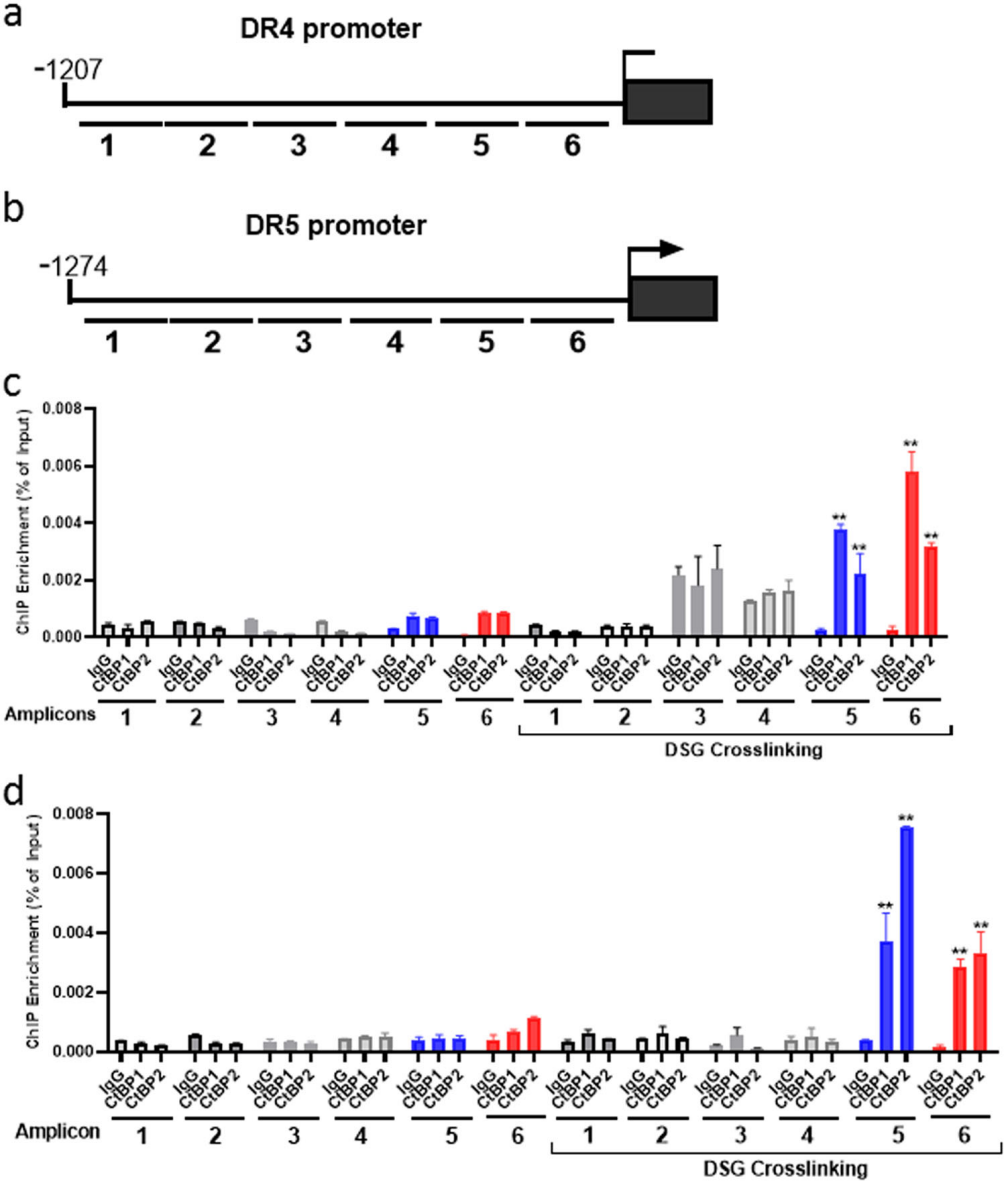
CtBP binds to DR4/5 promoters. **a** Cartoon showing the DR4 promoter. Black bars labeled 1–6 represent the location of PCR amplicons used in ChIP experiments. **b** Cartoon showing the DR5 promoter. Black bars labeled 1–6 represent the location of PCR amplicons used in ChIP experiments. **c** CtBP1/2 occupancy at the DR4 promoter in OVCA429 cells. ChIP was performed with OVCA429 chromatin, and PCR amplicons from (**a**) were used for ChIP, **p < 0.01 as compared with normal IgG group. **d** CtBP1/2 occupancy at the DR5 promoter in OVCA429 cells. ChIP was performed with OVCA429 chromatin, and PCR amplicons from (**b**) were used for ChIP, **p < 0.01 as compared with normal IgG group.

Considering that CtBP is a transcriptional corepressor, we performed ChIP assay to determine whether CtBP directly binds to the transcriptional regulatory regions of the DR4/5 genes in OVCA429 cells. To test the CtBP1/2 occupancy of the DR4/5 promoters, we designed six pairs of qPCR primers, spanning each promoter region, as illustrated in Fig. 5a, b. ChIP assay utilizing CtBP1 and CtBP2-specific antibodies (each antibody was non-cross reactive with the paralog; Supplementary Fig. S4) was performed and showed extremely weak signal for CtBP occupancy at either the DR4 or DR5 promoter (Fig. 5c, d). Considering that CtBP might form a regulatory complex with other factors, we included a protein–protein crosslinking step using DSG (disuccinimidyl glutarate). Under this condition, we identified significant occupancy of both CtBP1 and 2 within the promoter region proximal to the transcription start site of both the DR4 and 5 genes in OVCA429 cells (Fig. 5c, d).

### CtBP1 and CtBP2 functionally coordinate to repress DR4/5

**Fig. 6:**
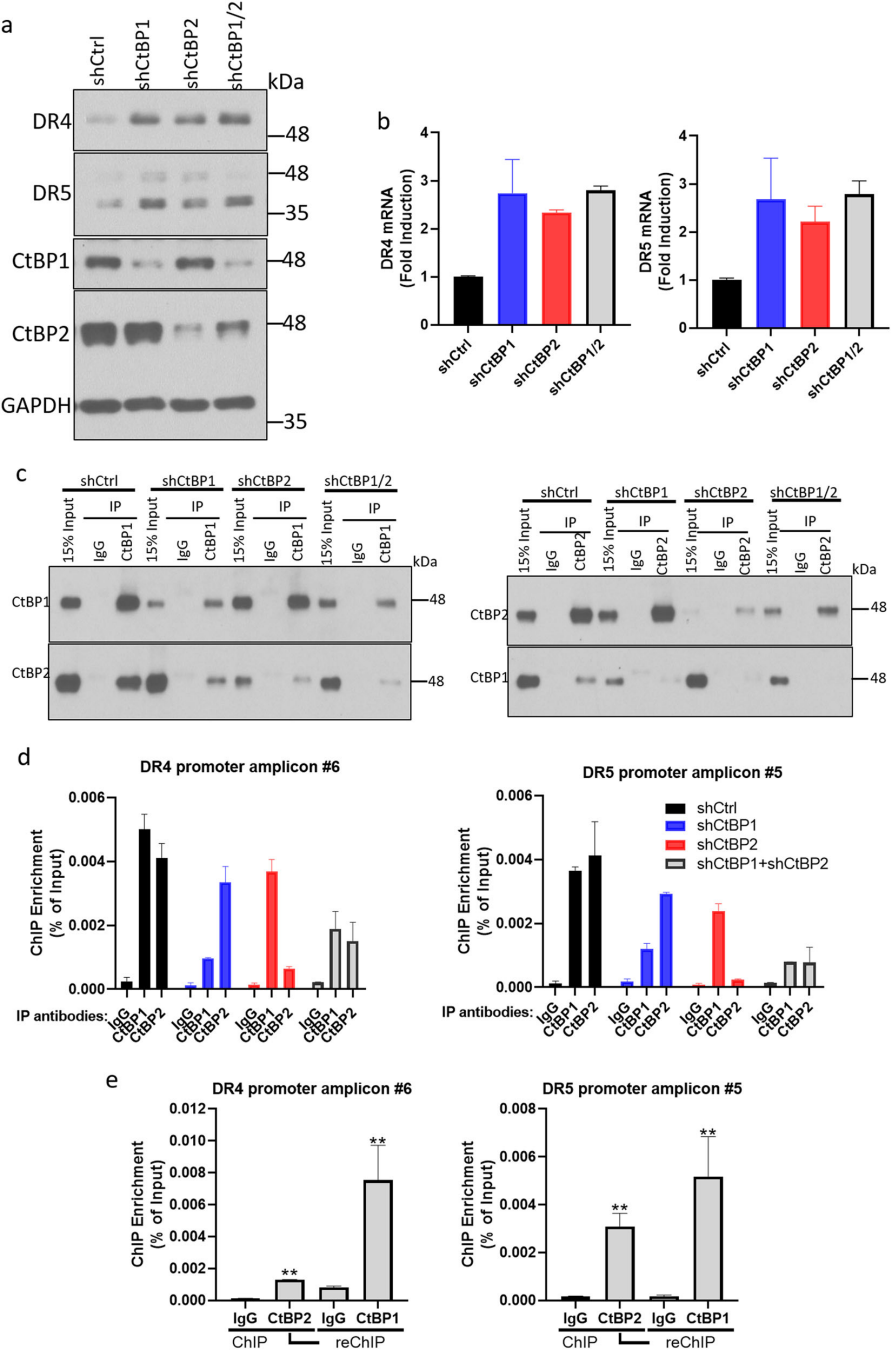
CtBP1 and CtBP2 coordinate to repress DR4/5. **a** Western blotting was performed to determine abundance of the indicated proteins in OVCA429 cells after infection with the indicated lentiviral shRNAs. **b** Fold induction of DR4/5 mRNAs in OVCA429 cells infected with the indicated lentiviral shRNAs was assessed by qPCR. **c** Western blotting to determine CtBP1/2 abundance in input and IP’s from ChIP experiments with OVCA429 cells when infected with indicated lentiviral shRNAs. **d** CtBP1/2 occupancy at DR4/5 promoters in OVCA429 cells. ChIP was performed with OVCA429 chromatin from cells in which CtBP1 or CtBP2 were knocked down with the indicated lentiviral shRNA’s. Indicated PCR amplicons were used for ChIP. **e** ChIP-reChIP assay showing CtBP1/2 occupancy at the indicated regions of the DR4/5 promotors. ChIP-reChIP was performed with OVCA429 chromatin and the first antibodies on the left hand side of the graphs, and second antibodies on the right hand side of each graph, **p < 0.01 as compared with normal IgG group.

Our data demonstrate that both CtBP1 and CtBP2 are present at the DR4/5 promoters, and that individually knocking down one or another led to upregulation of DR4/5 in OVCA429 cells. These findings raised a question of whether CtBP1/2 are dependent on each other to repress DR4/5. To address this possibility directly, we compared CtBP1/2 double knockdown OVCA429 cells against either CtBP1 or CtBP2 single knockdown cells and control knockdown cells. Western blotting and qPCR showed that DR4/5 proteins, as well as mRNAs, in CtBP1/2 double knockdown cells were induced to a similar level as that in single CtBP1 or CtBP2 knockdown cells (Fig. 6a, b), consistent with CtBP1 and CtBP2 repressing DR4/5 as a hetero-oligomeric complex, without redundancy between the paralogs for repression of DR4/5.

To address the mechanism underlying the requirement for both CtBP1 and 2 for DR4/5 repression, we tested the possibility that CtBP1 and 2 hetero-oligomerize in HGSOC cells by performing a co-IP with CtBP1 or 2 antibody and blotting for the paralog (Fig. 6c). Indeed, we observed robust hetero-oligomerization of CtBP1 and 2 by this method, suggesting that they could exist as a complex at target promoters, such as DR4/5 (Fig. 6c). To address this possibility directly, we performed a ChIP assay to test whether deficiency of CtBP1 or CtBP2 will dissociate the other paralog from DR4/5 promoter regions, but interestingly, knocking down CtBP1 or CtBP2 did not exclude the occupancy of the paralog from either promoter (Fig. 6d). However, ChIP-reChIP assay (performing a ChIP with first antibody and then dissociating and performing ChIP with a second antibody before analysis for bound promoter fragments) revealed that CtBP1 and CtBP2 are both physically present together at the same regions of DR4/5 promoters, consistent with their ability to coimmunoprecipitate from cell lysates, and consistent with the requirement for both to be present at the DR4/5 promoters for repression to occur (Fig. 6e). These results support CtBP1 and CtBP2 coordinate and cooperative regulation of DR4/5.

## Discussion

The prior findings of CtBP1/2 overexpression in ovarian cancer prompted us to investigate whether HGSOC cells exhibit dependency on CtBP. We identified CtBP1/2 as selective and direct repressors of DRs DR4 and DR5 in a cell-type dependent manner. CtBP1 and 2 coordinately controlled HGSOC cell apoptosis through death receptor/caspase 8 signaling that varied by cell as to TRAIL dependency. Most surprisingly, and in accordance with data showing both CtBP1 and CtBP2 amplification and overexpression in ovarian cancer, both paralogs were required simultaneously to maintain repression of DR4/5, suggesting they work coordinately, and indeed, as a heteroligomeric complex at the DR4/5 promoters.

Of note, reduction of CtBP1/2 by RNAi triggered caspase 8-dependent apoptosis in KURAMOCHI cells. Canonical TRAIL-induced apoptosis is mediated by activation of DR4/5, which signal to caspase 8 and the downstream caspase cascade. Recent studies have pointed out that intracellular aggregation of DR4/5 can also trigger caspase 8 activation, leading to ligand-independent, cell-autonomous apoptosis^37,39,45^. Indeed, we confirmed that induction of DR4, but not DR5, in KURAMOCHI cells by CtBP1/2 depletion governed caspase 8 activation, which did not require addition of any exogenous TRAIL. Knocking down CtBP did promote robust DR5 expression in other HGSOC cells, which was sufficient to activate caspase 8, but only after the addition of exogenous TRAIL. Of note, the heterogeneity in effect of CtBP knockdown on DR4/DR5 expression and TRAIL sensitization cannot be explained by variability of shRNA knockdown efficiency, as shCtBP1 and shCtBP2 effectively resulted in robust knockdown of both proteins across the spectrum of cell lines tested in this work. A key area for future investigation will be to determine why certain HGSOC lines can undergo cell-autonomous activation of DR signaling, while others require exogenous TRAIL to signal apoptosis. Given the many difficulties with therapeutic development of TRAIL and DR4/5 agonists^46^, our work could point the way to rational application of these agents in a precision approach to only those tumors where DR4 or DR5 are activated by CtBP inhibition, or those where TRAIL sensitization occurs secondary to CtBP inhibition.

Considering that CtBP proteins are transcriptional regulators, we performed ChIP assays to define the role of CtBP in regulation of DR4/5. As expected, both CtBP1 and CtBP2 were physically present at the promoter regions of DR4/5, and depletion of CtBP1/2 resulted in upregulation of DR4/5, indicating CtBP1/2 are both corepressors of DR4/5 expression.

It is conceivable that CtBP1/2 bind to other regulators to form a negative regulatory complex at those promoters. Future study is needed to elucidate the components of this complex. The regulation of DR4/5 genes by CtBP could also occur at multiple levels^47,48^. Analysis of clinical ovarian cancer samples as well as cell lines have revealed hypermethylation in the promoters of the DR4/5 genes^49–51^. Considering the phenotype of DR4/5 induction by CtBP inhibition depends on cell-type context, one possible explanation could be that full induction of DR4/5 requires removal or disruption of CtBP associated complexes and associated promoter hypermethylation. In certain cell lines, inhibition of CtBP1/2 is a prerequisite, but not sufficient to activate DR4/5, such as KURAMOCHI cells, where DR5 is not expressed, even after CtBP1/2 knockdown. To this point, it is very possible that the combination of targeting CtBP and demethylation of the promoter could be a powerful approach to induce DR4/5 expression and TRAIL-dependent or independent cancer cell death in HGSOC.

Since CtBP1/2 proteins share a high degree of homology, and both proteins are present in the nucleus, it was assumed that CtBP1/2 interchangeably modulate transcription. However, our data suggest that CtBP1/2 rely on each other in transcription suppression. First, double knockdown of CtBP1/2 did not bring DR4/5 expression to a higher level than that of individual CtBP1 or CtBP2 knockdown; second, CtBP1 knockdown induced DR4/5 expression without altering promoter occupancy of CtBP2; third, CtBP1 and 2 are stoichiometrically in complex together both in solution and at target promoters. Our study thus supports functional CtBP1/2 heterodimerization in transcriptional regulation, but to absolutely prove this point would require replacement of one paralog with the other genetically via genetic knock-in to determine if two copies of one of the paralogs is functionally equivalent to one copy of each. Of note, previous studies have shown that CtBP polymerization contributes to its regulatory effects, and CtBP dimerization is regulated by cellular NADH binding to its conserved dehydrogenase domain^52,53^. Pharmacologic reduction of NADH depolymerizes CtBP, resulting in induction of certain normally repressed target genes^54,55^. Since CtBP dimers can be disrupted not only by reduction in NADH level, but also by small molecule CtBP dehydrogenase inhibitors^56^, a promising approach for treatment of HGSOC and other CtBP dependent cancers that is currently under investigation may be combining small molecule CtBP inhibition with strategies that reduce cellular NADH level^57^.

Taken together, our results establish CtBP dependency in HGSOC via modulation of DR4/5. CtBP1/2 or a regulator of their activity could therefore be a promising target for cancer therapy.

## Supplementary information

Supplementary figure legends

Supplementary Fig. S1

Supplementary Fig. S2

Supplementary Fig. S3

Supplementary Fig. S4
