# Supplementary material for: CtBP determines ovarian cancer cell fate through repression of death receptors
Source: Cell Death Dis. 2020 Apr 24;11(4):286. doi: 10.1038/s41419-020-2455-7 (PMC7181866; doi:10.1038/s41419-020-2455-7)
Supplement: Supplementary file 1 — Supplementary figure legends [file 41419_2020_2455_MOESM1_ESM.docx]

**Supplementary figure legends**

**Supplementary Fig. S1**

**a & b**. KURAMOCHI cells were infected with indicated shRNAs (**a**) or transfected with indicated siRNAs (**b**) for 48 hours, endogenous TRAIL mRNA level was examined by qPCR, ***p<0.001 as compared with siCtrl group.

**c**. KURAMOCHI cells were treated with indicated siRNAs along with shRNAs, viable cells were counted by Trypan blue assay at indicated time points.

**Supplementary Fig. S2**

KURAMOCHI cells were infected with indicated shRNAs for 48 hours, then were treated with vehicle or TRAIL for 48 hours. Cell viability was assessed using a CCK-8 kit.

**Supplementary Fig. S3**

CAOV3 and HEY cells were infected with indicated shRNAs for 48 hours, DR4, DR5, CtBP1 and CtBP2 protein levels were examined by Western blotting.

**Supplementary Fig. S4**

Validating the specificity of CtBP antibodies. IP’s of lysates from OVSAHO, OVSAHO/CtBP1 KO, HCT116, and HCT116/CtBP2 KO cells were performed using the

indicated antibodies, followed by immunoblotting of washed IP’s with the indicated antibodies.
