# Supplementary figures and images for: CtBP determines ovarian cancer cell fate through repression of death receptors
Source: Cell Death Dis. 2020 Apr 24;11(4):286. doi: 10.1038/s41419-020-2455-7 (PMC7181866; doi:10.1038/s41419-020-2455-7)

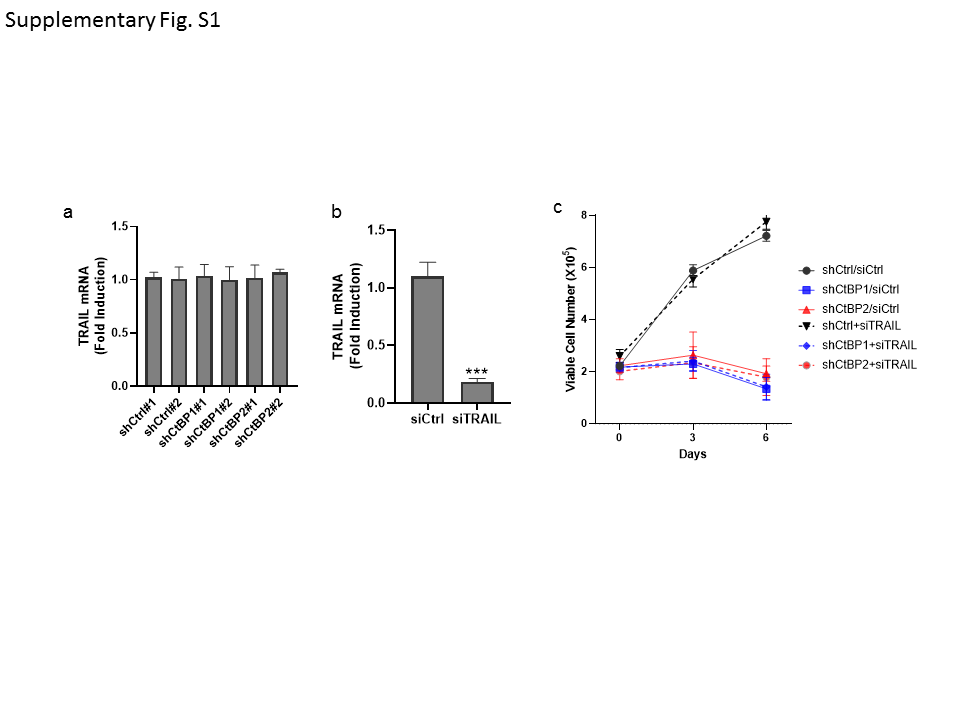

Supplement: Supplementary file 2 — Supplementary Fig. S1 [file 41419_2020_2455_MOESM2_ESM.tif]

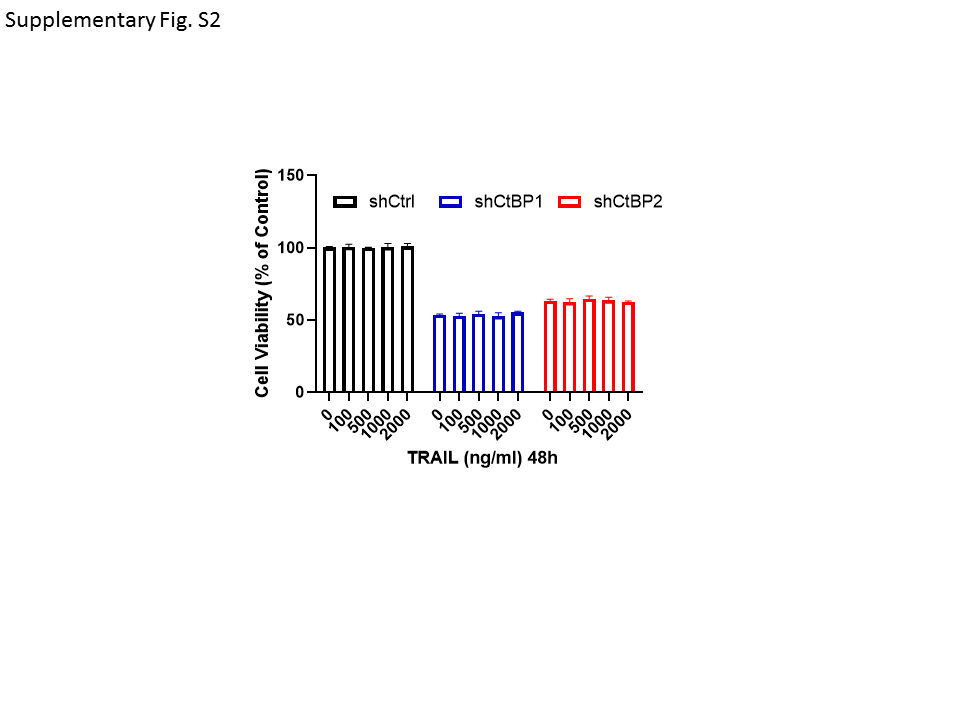

Supplement: Supplementary file 3 — Supplementary Fig. S2 [file 41419_2020_2455_MOESM3_ESM.tif]

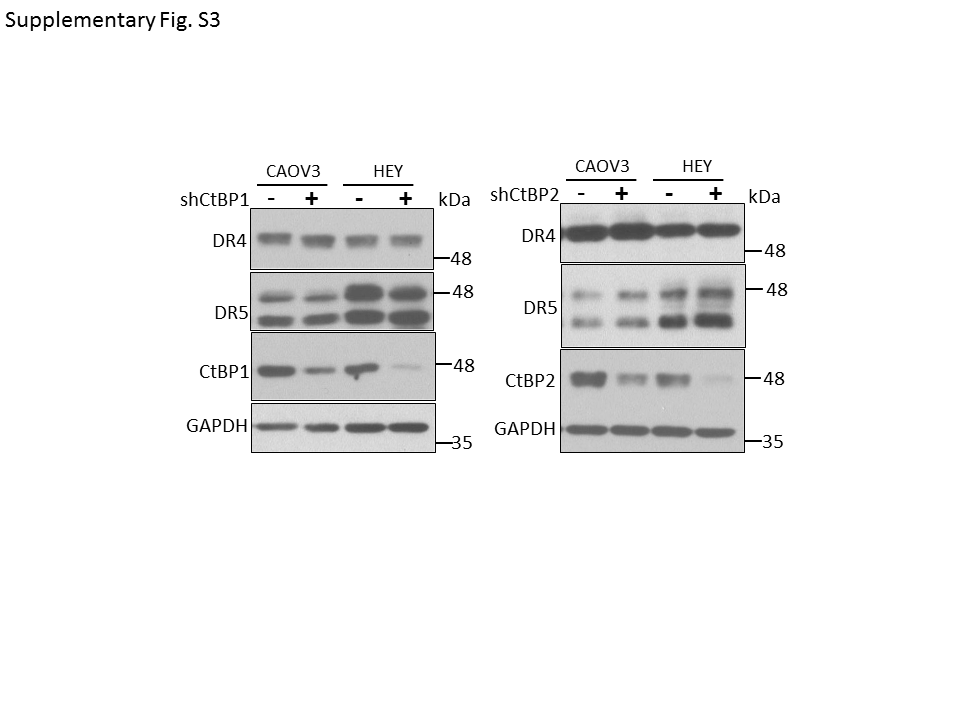

Supplement: Supplementary file 4 — Supplementary Fig. S3 [file 41419_2020_2455_MOESM4_ESM.tif]

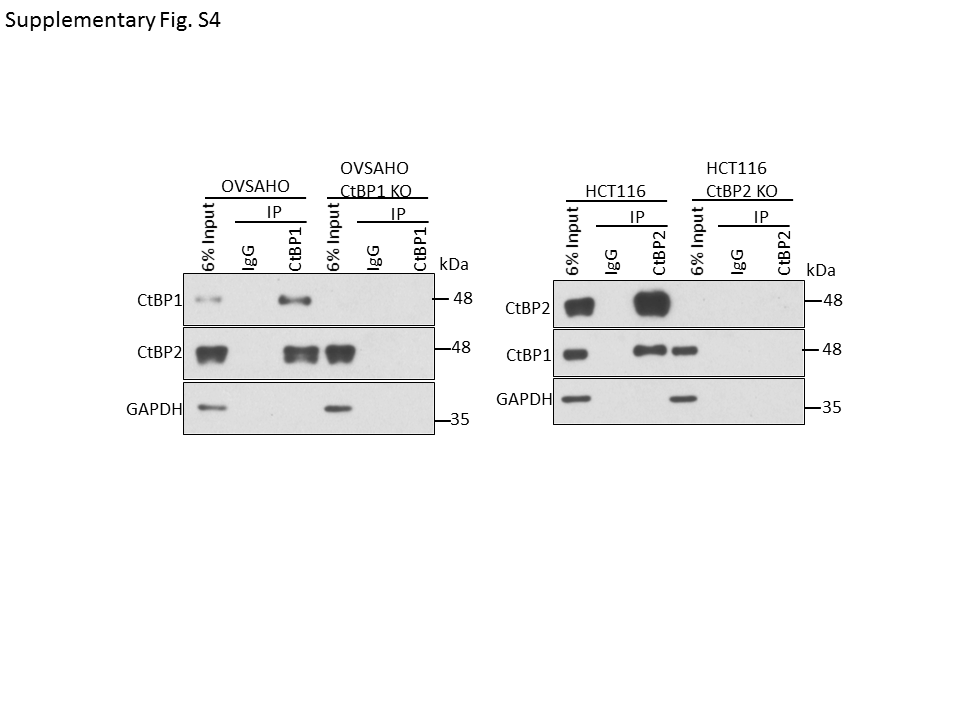

Supplement: Supplementary file 5 — Supplementary Fig. S4 [file 41419_2020_2455_MOESM5_ESM.tif]
